# Perspectives on model forecasts of the 2014–2015 Ebola epidemic in West Africa: lessons and the way forward

**DOI:** 10.1186/s12916-017-0811-y

**Published:** 2017-03-01

**Authors:** Gerardo Chowell, Cécile Viboud, Lone Simonsen, Stefano Merler, Alessandro Vespignani

**Affiliations:** 1 School of Public Health, Georgia State University, Atlanta, GA USA; 2 Division of International Epidemiology and Population Studies, Fogarty International Center, National Institutes of Health, Bethesda, MD USA; 3 Department of Public Health, University of Copenhagen, Copenhagen, Denmark; 4 Department of Global Health, George Washington University, Washington DC, USA; 5 Bruno Kessler Foundation, Trento, Italy; 6 Laboratory for the Modeling of Biological and Socio-technical Systems, Northeastern University, Boston, MA USA

**Keywords:** Ebola, West Africa, Epidemic model, Lessons learned, Disease forecast, Exponential growth, Sub-exponential growth, Polynomial growth, Data sharing

## Abstract

The unprecedented impact and modeling efforts associated with the 2014–2015 Ebola epidemic in West Africa provides a unique opportunity to document the performances and caveats of forecasting approaches used in near-real time for generating evidence and to guide policy. A number of international academic groups have developed and parameterized mathematical models of disease spread to forecast the trajectory of the outbreak. These modeling efforts often relied on limited epidemiological data to derive key transmission and severity parameters, which are needed to calibrate mechanistic models. Here, we provide a perspective on some of the challenges and lessons drawn from these efforts, focusing on (1) data availability and accuracy of early forecasts; (2) the ability of different models to capture the profile of early growth dynamics in local outbreaks and the importance of reactive behavior changes and case clustering; (3) challenges in forecasting the long-term epidemic impact very early in the outbreak; and (4) ways to move forward. We conclude that rapid availability of aggregated population-level data and detailed information on a subset of transmission chains is crucial to characterize transmission patterns, while ensemble-forecasting approaches could limit the uncertainty of any individual model. We believe that coordinated forecasting efforts, combined with rapid dissemination of disease predictions and underlying epidemiological data in shared online platforms, will be critical in optimizing the response to current and future infectious disease emergencies.

## Background

The 2014–2015 Ebola epidemic in West Africa represents one of the most important international public health challenges posed by an emerging infectious disease in the African continent in recent history. The unprecedented spread of the virus was facilitated by delays in the initial identification of the outbreak, compounded by a systemic lack of health infrastructure in the region, as well as economic, social and cultural factors that hampered effective implementation of control efforts [1, 2]. The official end of the epidemic, with a final tally of 28,610 reported probable infections and 11,308 deaths [3], offers a good opportunity to reflect on the lessons learned from the interdisciplinary efforts that guided the international response, particularly with regard to mathematical modeling.

Public health authorities are increasingly using mathematical and computational models in their decision-making processes during epidemic emergencies to generate forecasts of disease burden and compare intervention strategies [4]. This was particularly salient during the 2014–2015 Ebola epidemic, as a number of international academic groups developed mathematical models of disease spread to forecast the trajectory of the outbreak and guide the international response under different transmission and control scenarios [4]. These modeling efforts often relied on limited epidemiological data on key transmission and severity parameters for Ebola, which are needed to robustly calibrate mechanistic models. While a previous review article surveyed the characteristics, parameter estimates, and performance (accuracy) of 66 mathematical modeling studies published during the Ebola epidemic in West Africa [4], we provide here a perspective on some of the challenges, experiences, and lessons drawn from the forecasting efforts. In particular, most models overestimated the peak and final size of the outbreak, in part because of failure to account for reactive population behavior and the clustered nature of transmission [4]. We believe that a more complete understanding of the factors that led to cessation of Ebola transmission and the regional (rather than global) spread of this particular outbreak could help improve predictive modeling of current and future infectious disease emergencies.

## Data availability and early forecasts

**Fig. 1:**
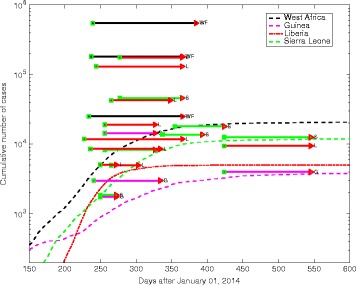
Observed trajectory of the Ebola epidemic in the three most affected countries of West Africa against predictions made in the midst of the outbreak. The colored horizontal lines represent model predictions for Guinea (G), Liberia (L), Sierra Leone (S), or all three countries combined (WF); the beginning of the line is when the prediction was made, whereas the end of the line marks the date the prediction is for (thus, shorter horizontal lines illustrate near-term predictions, while longer lines illustrate further time horizons). Data taken from JP Chretien’s Elife review [4]

During the early months of the Ebola epidemic in West Africa, up-to-date weekly Ebola case counts describing the course of the epidemic at the national level were made publicly available by the World Health Organization [5]. The data included probable and confirmed cases, as reported by local clinics and health districts. Lack of trained staff in epidemiology and disease- surveillance issues, varied levels of community participation, and limited telephone and internet services challenged Ebola reporting in the most affected countries [6]. Nationally aggregated data available within 1–2 weeks of occurrence was the primary publicly available source documenting the epidemic’s evolution. Many modelers around the world relied on this data source to estimate key transmission parameters and generate forecasts of morbidity and mortality impact (Fig. 1) [4]. To remedy the coarseness of publicly available data, parallel efforts from academic groups and private individuals were rapidly put in place to compile information from a variety of online sources and adjust publicly available data for reporting biases [7, 8].

During the early phase of the epidemic in West Africa, comprising the first 5–6 generations of disease transmission, the cumulative curve of Ebola case incidence suggested an exponential growth profile, indicating that transmission was sustained and the epidemic was becoming uncontrolled, with an estimated reproduction number of approximately 1.5–2.5 [4, 9–15]. Accordingly, early projections of the outbreak trajectory published in September 2014 indicated a pessimistic worst-case scenario, especially for long-term forecasts extending several months in advance [4, 13, 14].

**Fig. 2:**
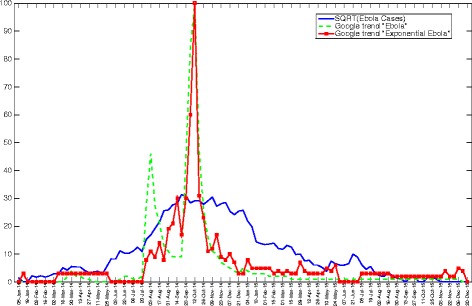
Google search volume – a signal that quantifies people’s web searches – for the phrase “Exponential Ebola” quickly surged and became popular during weeks 30–40, coinciding with the epidemic growth of reported cases in West Africa (Spearman’s rho = 0.64, *P* < 0.001). Interestingly, the popularity of this search term quickly plummeted after the epidemic peaked on week 40. For visualization purposes, the curve of the weekly number of new cases is square root transformed while the Google search trends indicate how often the terms “Ebola” and “Exponential Ebola” are searched for relative to the total number of searches (scale ranges from 0 to 100). The weekly series start with the first week in January 2014

The apparent exponential growth feature for the Ebola epidemic in West Africa rapidly disseminated among journalists and news media outlets [16]. In fact, Google search volume – a powerful signal that quantifies people’s web searches and attention – for the phrase “Exponential Ebola” quickly surged during weeks 30–40, roughly following the epidemic growth of reported cases in West Africa (Spearman’s rho = 0.64, *P* < 0.001; Fig. 2). The popularity of this search term quickly plummeted after the epidemic peaked on week 40 (Fig. 2).

## Moving beyond exponential growth assumptions

**Fig. 3:**
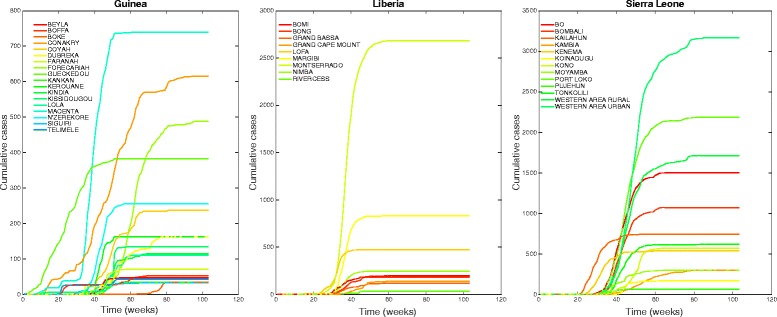
Representative time series of the cumulative number of weekly Ebola cases at the district level in Guinea, Sierra Leone, and Liberia. The district-level epidemics are spatially asynchronous and display an early growth phase that is more consistent with polynomial, rather than exponential, growth dynamics. The first week in the series ends on January 5, 2014

**Fig. 4:**
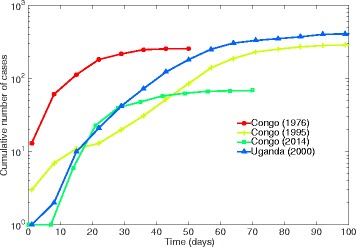
Cumulative curves of four past Ebola outbreaks in Congo (1976, 1995, 2014) [47–49] and Uganda (2000) [50]. These curves display rapid saturation in case growth within the first 3–4 generations of disease transmission, consistent with early sub-exponential growth dynamics

Refined sub-national epidemiological data at the level of counties or districts provided important clues about the actual pattern of Ebola spread. Such data only became publicly available in the World Health Organization patient database in November 2014 [3], only after the major surge in case incidence had subsided in the three most affected countries. The subnational epidemic curves displayed a remarkable level of spatial and temporal variability compared to aggregated national epidemic curves [5]. Indeed, local outbreaks were spatially asynchronous throughout the affected region (Fig. 3). Moreover, local-incidence growth patterns were characterized by rapid saturation after only a few generations of disease transmission, echoing past Ebola outbreaks but contrasting with the assumptions of homogeneous mixing models (Fig. 4).

At the district- or county-level, the first few generations of disease transmission in West Africa were largely characterized by sub-exponential growth dynamics of varying polynomial degrees [5, 17]. Even the Guinean district of Gueckedou, where the epidemic most likely originated, experienced a sub-exponential growth pattern by April 2014 (Fig. 3). Since this local outbreak took place before any large-scale attention or intervention measure was put in place, its growth patterns likely reflects the combined effects of reactive behavior changes and clustering of the contact network [5, 18, 19]. This departure from standard compartmental model theory affects estimates of transmission potential, projections of total epidemic sizes and the impact of interventions [20]. In particular, the effective reproduction number asymptomatically declines towards unity for sub-exponential growth outbreaks [21]. In contrast, for standard compartment models assuming exponential growth, the effective reproduction number remains invariant during the early phase of an epidemic, before susceptible depletion and interventions set in.

Sub-exponential growth patterns seen during the Ebola epidemic in West Africa are reminiscent of the HIV/AIDS epidemic in the US [22–24], another infectious disease transmitted by contact via infectious body fluids. In contrast, for an infection like influenza, which transmits readily through aerosols and droplets, epidemic growth is close to exponential, especially in pandemic situations [17]. The mechanisms that give rise to different epidemic growth profiles include features of the host and pathogen, including transmission route, individual behaviors, background immunity, and control interventions [25]. The relative importance of these mechanisms is difficult to quantify, and thus to model, in the absence of detailed information on fine-scale contact patterns early in the epidemic. In the case of Ebola, it is now thought that a combination of mechanisms were involved, including the social contact network, the heterogeneous susceptibility and infectivity of the population, and the reactive preventive behavior changes or mitigating measures as the population becomes gradually aware of the epidemic [5]. In particular, Ebola transmission chains tend to be spatially clustered within households, treatment facilities, and unsafe burials, as would be expected for a disease transmitted by close contact. Furthermore, Ebola-infected individuals are typically confined at home or in healthcare settings, particularly at the peak of infectiousness [5].

## A case for detailed agent-based models and more flexible compartmental models

The assumption of initial exponential growth is convenient to generate analytic expressions and estimates of the transmission potential (e.g., [26–28]). However, a necessary condition for validating a disease model is to be able to reproduce growth patterns that are consistent with observed epidemiological data [25], particularly if models are used for forecasting purposes.

**Fig. 5:**
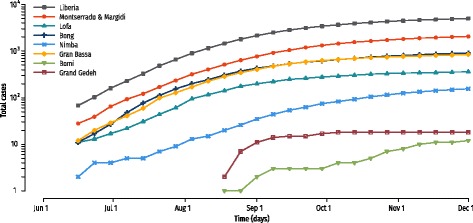
Mean of the cumulative number of cases for the most affected districts of Liberia (as predicted by an agent-based model in Liberia [30]); patterns are consistent with sub-exponential growth dynamics

With the increasing availability of data, computational power, and inference methods, agent-based modeling approaches have been increasingly sought to study the transmission dynamics and control of infectious diseases [25, 29]. The first individual-based simulation model for the Ebola epidemic in West Africa analyzed the situation in Liberia as a case study [30]. Uniquely resolved geotagged demographic information was compiled, along with population mobility data, the location of clinics and, later, Ebola treatment units to generate synthetic populations over which a disease process can be superimposed [30]. This agent-based model provided a realistic description of the epidemic and reproduced key features of the observational data, namely early sub-exponential growth and saturation after a few generations of disease transmission [30, 31] (Fig. 5). Later, this approach was relevant in assessing the effectiveness of interventions, pointing to the importance of contact tracing [30].

The agent-based model encoded two key epidemiological features of the Ebola epidemic, namely (1) high clustering of cases, as illustrated by a high proportion of secondary infections in households or extended households, and (2) modification of the social contact networks induced by isolation of cases in Ebola Treatment Units. Model projections compared well with observed transmission chains in West Africa, consistently showing that more than 70% of transmission events can result from the family or extended family members [31–33]. High clustering of transmission events results from the particular epidemiology of the disease, with most Ebola cases confined in households for a period of about 4 to 5 days prior to hospitalization, resulting in quick deviation from exponential growth [17]. Accordingly, mathematical models incorporating sub-exponential growth dynamics offered substantial improvements in forecasts of the trajectory and size of the epidemic [34], although they became available late in the outbreak.

## Transmission estimates and forecasts are challenging early on

As an outbreak unfolds in a population, public health authorities are interested in obtaining reliable estimates of the transmission potential of the infection and associated uncertainty, and how these estimates compare with those derived from past outbreaks. Phenomenological models that characterize the early epidemic growth phase with limited case data, together with information about the distribution of the generation interval of the disease, have proved useful to generate robust estimates of the effective reproduction number. This approach does not require explicitly modeling the mechanisms of disease transmission and control [21, 35, 36]; these methods are more suitable for outbreaks disseminating in large populations rather than confined to particular settings like hospitals, ships, or prisons [37–40]. Furthermore, with detailed information on transmission chains – describing who infects whom and typically derived from contract-tracing efforts – it becomes possible to generate more precise estimates of the reproduction number. In particular, one can assess changes in transmission by disease generation and pinpoint individuals who may contribute disproportionately to transmission (e.g., SARS [37], MERS [37], Ebola [31, 32, 41, 42]).

Another key quantity of interest for public health authorities early on is how large the epidemic will be. This requires predictions of outbreak trajectory a few weeks to months ahead, which are considered short- to long-term forecasts (more akin to climate rather than weather forecasts). An important caveat of such disease forecasts is that the magnitude of interventions and reactive behavior changes cannot be fully predicted, especially when there is little prior information from past outbreaks to rely on. This goes beyond the uncertainty associated with the underlying model structure and can really only be addressed through sensitivity analyses considering different epidemiological scenarios. Thus, early forecasting efforts that have more than a few weeks’ time horizon should really be considered as scenario evaluations rather than projections per se.

## Looking forward

The nationally aggregated Ebola epidemic data available during the first few months of the West African outbreak missed the important patterns observed in local data regarding transmission dynamics. This highlights the need to exercise caution when analyzing and interpreting spatially aggregated transmission patterns, especially when limited information is available on prior large-scale outbreaks. Conversely, dire estimates of Ebola epidemic size derived early on from homogeneous mixing models were likely the catalyst for a comprehensive and strong international public health response to eliminate the epidemic. Thus, these early estimates had an important role for advocacy.

**Fig. 6:**
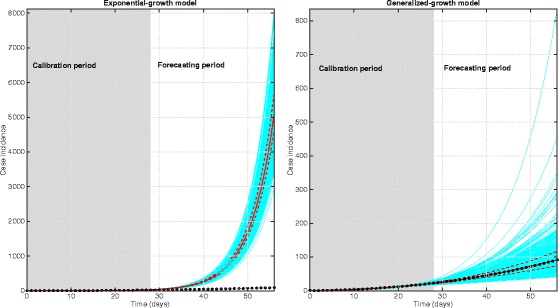
Forecasting early epidemic growth phase data featuring sub-exponential growth dynamics using a classic exponential growth model (*left*) and the generalized growth model (right). The shaded region corresponds to the model calibration period and the non-shaded area corresponds to the forecasting period. Circles correspond to the case-series data. The blue curves correspond to the ensemble of epidemic forecasts. The red solid and dashed lines correspond to the median and interquartile range computed from the ensemble of forecasts, respectively. This figure illustrates how extrapolations of epidemic impact from the early growth trend in case incidence of an epidemic are subject to both model and data uncertainty. Transmission models calibrated using a few data points of the early phase of an infectious disease outbreak assuming exponential growth epidemic dynamics, such as the widely used SIR-type compartmental models, are unable to predict anything other than an exponentially growing epidemic in the absence of susceptible depletion, interventions or behavior changes, leading to great overestimation of cumulative case burden. More flexible transmission models, such as the generalized growth model, capture a wider range of epidemic growth profiles, ranging from sub-exponential to exponential growth dynamics. Please note the figures are on a different scale

Extrapolations of epidemic impact from the early growth epidemic phase are subject to model, data, and behavioral uncertainty [43]. Indeed, based on epidemic data during the early epidemic growth phase, it is possible that (1) the data do not convey sufficient information to reliably ascertain the profile of epidemic growth and assess transmission potential and final size, even in the absence of interventions, and that (2) key aspects of transmission dynamics are not captured by the model (e.g., the model assumes a fixed type of epidemic growth). Transmission models that predict exponential growth can greatly overestimate epidemic size [4] without accounting for the mitigating effects of interventions or behavior changes (Fig. 6). More flexible models should be better equipped to fit the early growth dynamics of an epidemic process and provide more realistic uncertainty bounds for short- and long-term epidemic forecasts [17, 34]. Simple models incorporating generalized growth features have proved useful to characterize the early epidemic growth dynamics [17] and provide a starting point for characterizing epidemic growth and forecasting epidemic impact (e.g., epidemic size) [34]. The phenomenological models do not require a large amount of data; indeed, accurate assessment of the growth profile can be achieved within the first five disease generations (with 5–10 weeks’ worth of weekly district-level incidences) across a range of pathogens. As the epidemic unfolds and more data become available about transmission chains, detailed mechanistic models can be developed to make specific inferences about the contribution of different transmission sources (e.g., hospital, funeral, community) and quantify the effectiveness of behavioral changes and control interventions [30, 31, 44].

## Conclusion

The ability of mathematical modelers to generate useful disease forecasts in real time depends heavily on knowledge of the transmission process to guide model design and on the timely availability of data for model calibration. Key model ingredients include (1) epidemiological datasets, including case series describing the trajectory of the outbreak, to calibrate the baseline transmission characteristics of the outbreak of interest; (2) knowledge of the relevant modes of transmission (e.g., close contact, droplet, airborne), relevant transmission settings (e.g., hospital, school, funeral, community), and mobility patterns to design appropriate spatial structures and contact networks; and (3) the natural history of the disease, including latent, incubation, and infectious periods as well as information on the frequency of asymptomatic, mild, and symptomatic infections and their associated infectiousness. Looking back, early in the West African outbreak, there was a good amount of information on natural history parameters and transmission routes from past outbreaks, but the importance of mobility and contact networks was unclear, since all prior outbreaks were highly restricted geographically and did not involve large treatment facilities. These uncertainties could have been resolved more rapidly than they were if detailed transmission chains had been available earlier [45] (in fact, the earliest transmission chains were published in October 2014 and January 2015 for outbreaks in Nigeria [42] and Guinea [32], respectively).

The cautionary tale of Ebola, with its early pessimistic predictions, is not unique to severe diseases. Clustering of contact networks, saturation effects, local burnouts, and behavioral changes are common to many diseases. Deviation from simple exponential behavior can also be expected in diseases with a seasonal component, mediated by the vector life cycle, such as the Chikungunya and Zika virus epidemics. While, in many cases, the lack of data might not serve more elaborate models, the need for a portfolio of models that allow for deviations from the standard theory is extremely important. Such models should span the gamut of complexity, from highly abstracted phenomenological models (best when little data) to compartmental models allowing for behavior changes or clustered transmission, to more complex and highly detailed agent-based models. Looking to weather forecasts for guidance, a field with a well-established history of predictive approaches relying on real-time modeling analyses of multiple layers of data streams, policymakers will want to rely on ensemble model predictions rather than on any individual approach. Ensemble model predictions provide a broader and more accurate picture of the possible evolution of an emerging outbreak and, in turn, offer more solid guidance for control interventions. None of these modeling approaches are feasible without timely sharing of high-resolution epidemiological data and collaboration to interpret early data on transmissibility and severity [46]. This point was made in 2003 during the SARS crisis, but data sharing still has a long way to go as was evident in the 2014 Ebola crisis, and more recently in the Zika outbreak. As we look to the future, we must envision coordinated modeling and forecasting efforts facilitated though interactive website platforms and involving multiple research groups. Only in this way can individual groups, in real-time, readily share their approaches and results relying on consistent data sources and adequately documented methods, receive peer feedback, and disseminate collective results in joint publications.
